# DMM Prize 2018 winner: Wenqing Zhou

**DOI:** 10.1242/dmm.039586

**Published:** 2019-03-26

**Authors:** Monica J. Justice

**Affiliations:** Program in Genetics and Genome Biology, The Hospital for Sick Children, and Department of Molecular Genetics, The University of Toronto, Toronto, ON M5G 0A4, Canada

## Abstract

Disease Models & Mechanisms (DMM) is delighted to announce that the winner of the DMM Prize 2018 is Wenqing Zhou, for her paper entitled ‘Neutrophil-specific knockout demonstrates a role for mitochondria in regulating neutrophil motility in zebrafish’ (Zhou et al., 2018a). The prize of $1000 is awarded to the first author of the paper that is judged by the journal's editors to be the most outstanding contribution to the journal that year. To be considered for the prize, the first author must be a student or a postdoc of no more than 5 years standing.


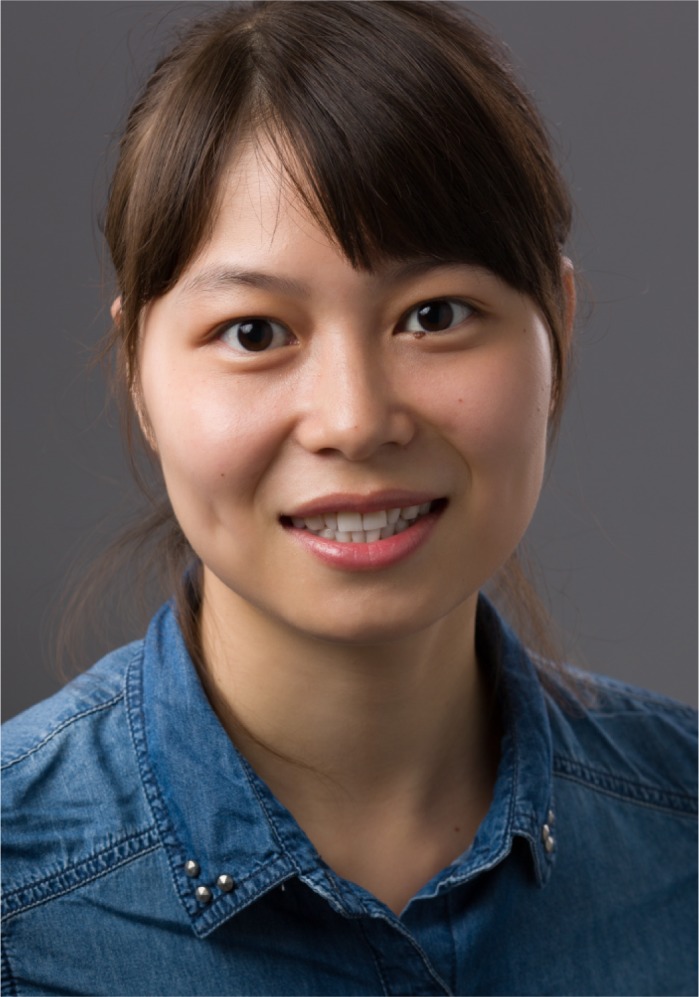


Wenqing Zhou

## Outstanding contribution

Wenqing Zhou grew up in a small town in South Central China and received her bachelor’s degree in biotechnology from Central South University, China. In the third year of undergraduate study, she joined Dr Xueduan Liu's lab, where she learned to isolate bacterial strains from the environment and utilize those strains to increase the metal extraction efficiency from mineral. During that time, she was deeply attracted by those ‘tiny but magic microbes’.

After graduation, in 2011, Wenqing joined the master's program at the Institute of Microbiology, Chinese Academy of Sciences, and began to study the antibiotic resistance of pathogens with the guidance of Dr Jie Feng. *Streptococcus pneumoniae*, one of the most common pathogens in the respiratory tract, is widely resistant to macrolide antibiotics because of the dissemination of transposon Tn2010, which carries two different macrolide-resistance genes. She identified that transformation is the predominant way to transfer Tn2010 between *S. pneumoniae*, and that the acquisition of Tn2010 has a negligible fitness cost, which may explain the widespread distribution of the transposon (Zhou et al., 2014).

The experience in Dr Feng's lab made her understand what research is, and she really enjoyed the process, so she decided to pursue an academic career. After graduation, she began her PhD under the direction of Dr Qing Deng at Purdue University, IN, USA, in 2014. In Dr Deng's lab, her first project was to determine the role of microRNA in the response of neutrophils to inflammation. She identified that miR-223 in epithelial cells regulates neutrophilic inflammation. miR-223 is known as a myeloid-enriched microRNA, and its expression is highest in neutrophils. However, she found that the augmented neutrophilic inflammation in miR-223-deficient zebrafish is mainly due to the over-activation of NF-κB in the basal layer of the surface epithelium. The intrinsic regulation of NF-κB in epithelial cells by miR-223 was further confirmed in human cells. This work provided a direct connection between miR-223 and the canonical NF-κB pathway, and highlighted an overlooked relevance of epithelial cells in dampening neutrophil activation (Zhou et al., 2018b).

Neutrophils are fast-moving cells and primarily rely on glycolysis for adenosine triphosphate (ATP) availability. Whether mitochondria regulate neutrophil motility *in vivo* remained obscure. Wenqing's second project focused on mitochondria and neutrophil migration. She optimized the original method developed by Dr Len Zon's group (Ablain et al., 2015) and established a gateway system harboring the CRISPR/Cas9 elements for tissue-specific knockout to disrupt mitochondrial function genetically in zebrafish (Zhou et al., 2018a). With this system, she found that neutrophil-specific disruption of mitochondrial DNA polymerase, *polg*, significantly reduces the velocity of neutrophil interstitial migration. In addition, inhibiting the mitochondrial electron transport chain or the enzymes that reduce mitochondrial reactive oxygen species (ROS) also inhibited neutrophil motility. Furthermore, the research demonstrated that the reduced cell motility resulting from neutrophil-specific knockout of *sod1* was rescued by *sod1* mRNA overexpression or by treating with scavengers of ROS. Together, their work established the first *in vivo* evidence that mitochondria regulate neutrophil motility, and provided insights into immune deficiency seen in patients with primary mitochondrial disorders. Technically, the group has used the tissue-specific-knockout approach to discover the function of genes, especially those that are developmentally essential, in neutrophils. This is the first successful example of using this technique to make scientific discoveries. They have further demonstrated the specificity of the knockout by mRNA and chemical rescue (Zhou et al., 2018a).

**Box 1. DMM Prize 2018 shortlist**

**Winner:**

**Neutrophil-specific knockout demonstrates a role for mitochondria in regulating neutrophil motility in zebrafish.**

Wenqing Zhou, Lingyan Cao, Jacob Jeffries, Xiaoguang Zhu, Christopher J. Staiger and Qing Deng.

Disease Models & Mechanisms (2018) 11, dmm033027. doi:10.1242/dmm.033027.

**Also shortlisted by our Editor team:**

**Bone marrow transplantation corrects haemolytic anaemia in a novel ENU mutagenesis mouse model of TPI deficiency.**

Ashlee J. Conway, Fiona C. Brown, Elinor J. Hortle, Gaetan Burgio, Simon J. Foote, Craig J. Morton, Stephen M. Jane and David J. Curtis.

Disease Models & Mechanisms (2018) 11, dmm034678. doi:10.1242/dmm.034678.

***Drosophila melanogaster*as a function-based high-throughput screening model for antinephrolithiasis agents in kidney stone patients.**

Sohrab N. Ali, Thamara K. Dayarathna, Aymon N. Ali, Tijani Osumah, Mohamed Ahmed, Tyler T. Cooper, Nicholas E. Power, Dongxing Zhang, Dajung Kim, Rachel Kim, Andre St. Amant, Jinqiang Hou, Thomas Tailly, Jun Yang, Len Luyt, Paul A. Spagnuolo, Jeremy P. Burton, Hassan Razvi and Hon S. Leong.

Disease Models & Mechanisms (2018) 11, dmm035873. doi:10.1242/dmm.035873.

**A novel rabbit model of Duchenne muscular dystrophy generated by CRISPR/Cas9.**

Tingting Sui, Yeh Siang Lau, Di Liu, Tingjun Liu, Li Xu, Yandi Gao, Liangxue Lai, Zhanjun Li, Renzhi Han.

Disease Models & Mechanisms (2018) 11, dmm032201. doi:10.1242/dmm.032201.

**The class I myosin MYO1D binds to lipid and protects against colitis.**

William McAlpine, Kuan-wen Wang, Jin Huk Choi, Miguel San Miguel, Sarah Grace McAlpine, Jamie Russell, Sara Ludwig, Xiaohong Li, Miao Tang, Xiaoming Zhan, Mihwa Choi, Tao Wang, Chun Hui Bu, Anne R. Murray, Eva Marie Y. Moresco, Emre E. Turer and Bruce Beutler.

Disease Models & Mechanisms (2018) 11, dmm035923. doi:10.1242/dmm.035923.

**Nmnat mitigates sensory dysfunction in a*Drosophila*model of paclitaxel-induced peripheral neuropathy.**

Jennifer M. Brazill, Beverley Cruz, Yi Zhu and R. Grace Zhai.

Disease Models & Mechanisms (2018) 11, dmm032938. doi:10.1242/dmm.032938.

**CRISPR-Cas9 human gene replacement and phenomic characterization in*Caenorhabditis elegans*to understand the functional conservation of human genes and decipher variants of uncertain significance.**

Troy A. McDiarmid, Vinci Au, Aaron D. Loewen, Joseph Liang, Kota Mizumoto, Donald G. Moerman and Catharine H. Rankin.

Disease Models & Mechanisms (2018) 11, dmm036517. doi:10.1242/dmm.036517.

**Spliceosomal components protect embryonic neurons from R-loop-mediated DNA damage and apoptosis.**

Shelly Sorrells, Sara Nik, Mattie Casey, Rosannah C. Cameron, Harold Truong, Cristhian Toruno, Michelle Gulfo, Albert Lowe, Cicely Jette, Rodney A. Stewart and Teresa V. Bowman.

Disease Models & Mechanisms (2018) 11, dmm031583. doi:10.1242/dmm.031583.

**Cancer modeling by transgene electroporation in adult zebrafish (TEAZ).**

Scott J. Callahan, Stephanie Tepan, Yan M. Zhang, Helen Lindsay, Alexa Burger, Nathaniel R. Campbell, Isabella S. Kim, Travis J. Hollmann, Lorenz Studer, Christian Mosimann and Richard M. White.

Disease Models & Mechanisms (2018) 11, dmm034561. doi:10.1242/dmm.034561.

**Cooperation of loss of*NKX3.1*and inflammation in prostate cancer initiation.**

Clémentine Le Magnen, Renu K. Virk, Aditya Dutta, Jaime Yeji Kim, Sukanya Panja, Zoila A. Lopez-Bujanda, Andrea Califano, Charles G. Drake, Antonina Mitrofanova and Cory Abate-Shen.

Disease Models & Mechanisms (2018) 11, dmm035139. doi:10.1242/dmm.035139.

Currently, Wenqing is characterizing the stable zebrafish lines that she generated with neutrophil-specific knockout, and trying to provide a full understanding of the pathway regulated by mitochondria in neutrophil migration. She will finish the work in the spring of 2019 and start to look for a postdoctoral position.

## References

[DMM039586C4] Ablain J., Durand E. M., Yang S., Zhou Y. and Zon L. I. (2015). A CRISPR/Cas9 vector system for tissue-specific gene disruption in zebrafish. *Dev. Cell* 32, 756-764. 10.1016/j.devcel.2015.01.032 25752963 PMC4379706

[DMM039586C1] Zhou W., Yao K., Zhang G., Yang Y., Li Y., Lv Y. and Feng J. (2014). Mechanism for transfer of transposon Tn*2010* carrying macrolide resistance genes in *Streptococcus pneumoniae* and its effects on genome evolution. *J. Antimicrob. Chemother.* 69, 1470-1473. 10.1093/jac/dku019 24532683

[DMM039586C2] Zhou W., Cao L., Jeffries J., Zhu X., Staiger C. J. and Deng Q. (2018a). Neutrophil-specific knockout demonstrates a role for mitochondria in regulating neutrophil motility in zebrafish. *Dis. Model. Mech.* 11, dmm033027 10.1242/dmm.033027 29590639 PMC5897731

[DMM039586C3] Zhou W., Pal A. S., Hsu A. Y.-H., Gurol T., Zhu X., Wirbisky-Hershberger S. E., Freeman J. L., Kasinski A. L. and Deng Q. (2018b). MicroRNA-223 suppresses the canonical NF-kB pathway in basal keratinocytes to dampen neutrophilic inflammation. *Cell Rep.* 22, 1810-1823. 10.1016/j.celrep.2018.01.058 29444433 PMC5839657

